# A novel cardiovirus in wild rats

**DOI:** 10.1186/s12985-018-0968-9

**Published:** 2018-03-27

**Authors:** Yan Wang, Jing Zhao, Min Zheng, Zhijian Liu, Wang Li, Xingli Fu, Yuan Lin, Jiaqi Yuan, Jieji Zhao, Quan Shen, Xiaochun Wang, Hua Wang, Shixing Yang

**Affiliations:** 1 School of Medicine, Jiangsu University, 301 Xuefu Road, Zhenjiang, Jiangsu 212013 People’s Republic of China; 2 Department of Gynecology, Rizhao Maternity& Infant Health Hospital, Rizhao, Shandong 276800 People’s Republic of China; 3 School of Basic Medical Sciences, Ningxia Medical University, Yinchuan, Ningxia 750000 People’s Republic of China

**Keywords:** *Cardiovirus* genus, Viral metagenomics, Genomic structure, Phylogenetic analysis

## Abstract

Cardioviruses cause severe illnesses in rodents and humans. In recent years, novel cardioviruses have been frequently found, which promoted further studies of the genetic diversity of cardioviruses. Using viral metagenomics, we genetically characterized a novel cardiovirus (named SX1) from wild rat feces. The genomic structure of SX1 shared similar features with those of the Theiler’s murine encephalomyelitis viruses, including a leader protein, four structural proteins and seven non-structural proteins. Phylogenetic analysis based on both structural proteins and non-structural proteins coding regions showed that SX1 was formed into a separate branch, being located between the branches of Theiler’s murine encephalomyelitis viruses and Thera viruses. Variable resides presented in the Ser/Thr rich domain of L protein, VP1 loops, and VP2 puffs distinguished SX1 from Theiler’s murine encephalomyelitis viruses, suggesting the different antigenicity and pathogenicity of SX1.

## Findings

Cardioviruses is a genus of picornaviruses that cause severe illnesses in rodents and human [1–4]. The genus *Cardiovirus* includes three species, namely *Cardiovirus A*, *Cardiovirus B, and Cardiovirus C*. *Cardiovirus A* has only one member, encephalomycarditis virus (EMCV), which causes rat encephalitis and myocarditis [5, 6]; *Cardiovirus C* is a novel cardiovirus identified in laboratory rats and *Rattus norvegicus* [7]; *Cardiovirus B* is composed of Theiler’s murine encephalomyelitis virus (TMEV), Vilyuisk human encephalomyelitis virus (VHEV), Thera virus (TRV), and Saffold virus (SAFV), where VHEV and SAFV can infect humans and cause encephalomyelitis, acute gastroenteritis and so on [1, 8, 9]. TMEV and TRV mainly infect mice and cause neurological diseases [3, 10, 11]. TMEVs were originally isolated from colony-bred mice that developed spontaneous paralysis in the early 1930s [12]. Now it is reported that TMEVs mainly cause asymptomatic infections in mice, and in rare cases, neurological symptoms featured with early poliomyelitis or late demyelinating disease [13]. TRVs were firstly isolated from sentinel rats housed with TMEV-seropositive rats in Japan in 2002 [14]. In 2008, a novel isolate of TRV was detected in the feces of rats [3]. Till now, there is no report about the association between TRV and diseases in rats. Using virus metagenomics method, we detected a novel cardiovirus in the feces of wild rats and characterized the complete genome. The novel cardiovirus was named SX1 and its genomic sequence was submitted to GenBank with accession no. MF172923.

In order to investigate rat intestinal virome, 40 intestinal content samples were collected from wild *M. longicaudus* rats captured by the Chinese Center for Disease Control and Prevention in Taizhou City from three districts of Taizhou City including Taixing (*n* = 15), Gaogang (n = 15), and Hailing (*n* = 10) from June to August in 2014. All of the wild rats were adults and the exact ages were unknown.

Viral metagenomics method was used to identify viral sequences in these samples. Four separate pools were randomly generated, each of which contained 10 fecal specimens. Briefly, fecal samples were suspended in Dulbecco’s phosphate-buffered saline (DPBS). After low speed centrifugation and filtration, the samples were treated with DNase and RNase to reduce levels of rat nucleic acids while viral genome within viral capsid was protected from digestion [15]. Four libraries were then constructed using Nextera XT DNA Sample Preparation Kit (Illumina) and sequenced using the Miseq Illumina platform with 250 bases paired ends with a distinct molecular tag for each pool. Bioinformatics analysis was performed according to a previous study [15].

**Fig. 1:**
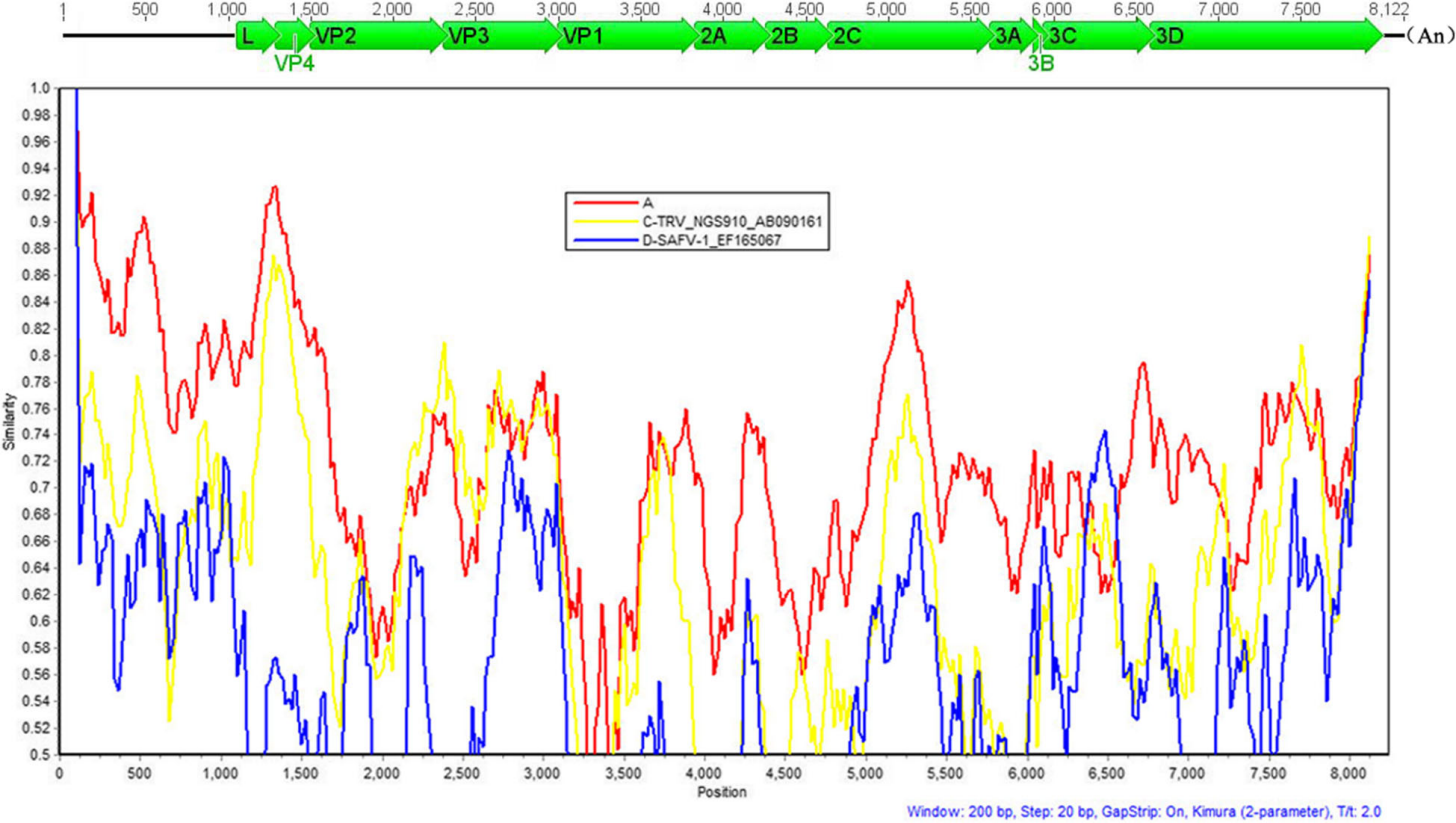
Genomic structure of SX1 and similarity analysis basing on full genome nucleotide sequences. The similarity analysis of complete genomes was calculated by Simplot 3.5.1 with a sliding window of 200 nucleotides moving in steps of 20 nucleotides. All positions with gaps were deleted, and the nucleotide similarity was plotted using the JC model of nucleotide substitution. All sequences were divided into four groups, group A marked with red line included 6 TMEV strains (GenBank accession no. KJ191558, EU718733, M16020, EU718732, M20562, and M20301), group B just included SX1 as query, group C marked with yellow line only had one TRV strain (GenBank accession no. AB090161), group D marked with blue line only had one SAFV strain (GenBank accession no. EF165067)

Miseq Illumina sequencing generated 16,832 unique reads which contained abundant viral sequences reads based on BLASTx search. Numbers of sequence reads showing similarities to known viruses included 2097 in pool-1, 5375 unique reads in pool-2, 7953 reads in pool-3, and 1407 reads in pool-4. Pool-3 contained 417 sequence reads showed significant sequence similarities to Theilovirus. PCR was then performed to bridge the gaps between sequence contigs and Rapid Amplification of cDNA Ends (RACE) amplification was used to acquire the complete genome. The resulted complete genome of SX1 is consisted of 8102 bp, in addition to a poly (A) at 3′ terminus. A single 2304-amino acid polyprotein is found which is composed by 12 proteins, including a leader peptide (L), four structural proteins (VP1, VP2, VP3, and VP4), and seven nonstructural proteins (2A, 2B, 2C, 3A, 3B, 3C, and 3D). Using the SimPlot software [16], the sequence similarity and putative homologous recombination analyses between SX1 and the other strains of *Cardiovirus B* were performed. Results showed that SX1 had higher sequence similarity to TMEVs than TRVs and SAFVs (Fig. 1).

**Fig. 2:**
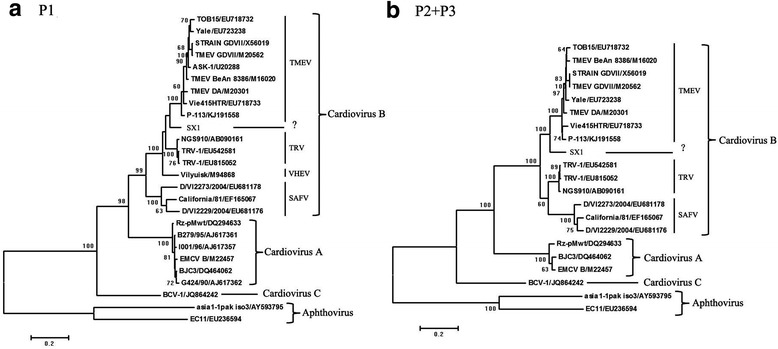
Phylogenetic relationships among cardioviruses based on the alignment of the nucleotide sequences of the P1, and P2 + P3 coding regions. **a** Phylogenetic analysis based on the P1 region. **b** Phylogenetic analysis based on the P2 and P3 regions. An unrooted tree was reconstructed using the neighbor-joining algorithm implemented in MEGA 6.0. Strain names and GenBank accession numbers of cardioviruses were shown. Scale bar indicated nucleotide substitution per site. Bootstrap valued (based on 1000 replicates) for each node are given

In order to further determine the genetic relationship of SX1 with the other members of Cardioviruses, phylogenetic analysis based on both the structural protein region (P1) and the nonstructural protein regions (P2, and P3) showed that SX1 showed that SX1 formed a separate branch (Fig. 2a and b). Sequence comparison between SX1 and TMEVs, TRVs, SAFV, and VHEV indicated the amino acid sequence identities of P1 and P2 + P3 were 63–85% and 48–84%, respectively. The P1 region of SX1 shared the highest amino acid sequence identity (85%) with a TRV strain (GenBank accession no. EU542581), which was isolated from infected rats [3]. The P2 + P3 region of SX1 shared the highest amino acids sequence identity (84%) with a TMEV strain (GenBank assession no. KJ191558), which was isolated from the wild boars. Based on the VP1 region, SX1 shared the amino acid sequence identities < 77.9% with TMEVs and TRVs, while the identities among the members within TMEVs and TRVs were 87.7–94.6%, 92.7–97.1%, respectively. Although SX1 failed to meet the criteria that a new Cardiovirus species should have < 60% aa identity of P1 comparing with the other known Cardiovirus species according to the International Committee on Taxonomy of Viruses, the results of phylogenetic analysis indicated that SX1 might be a novel type of Cardiovirus.

**Fig. 3:**
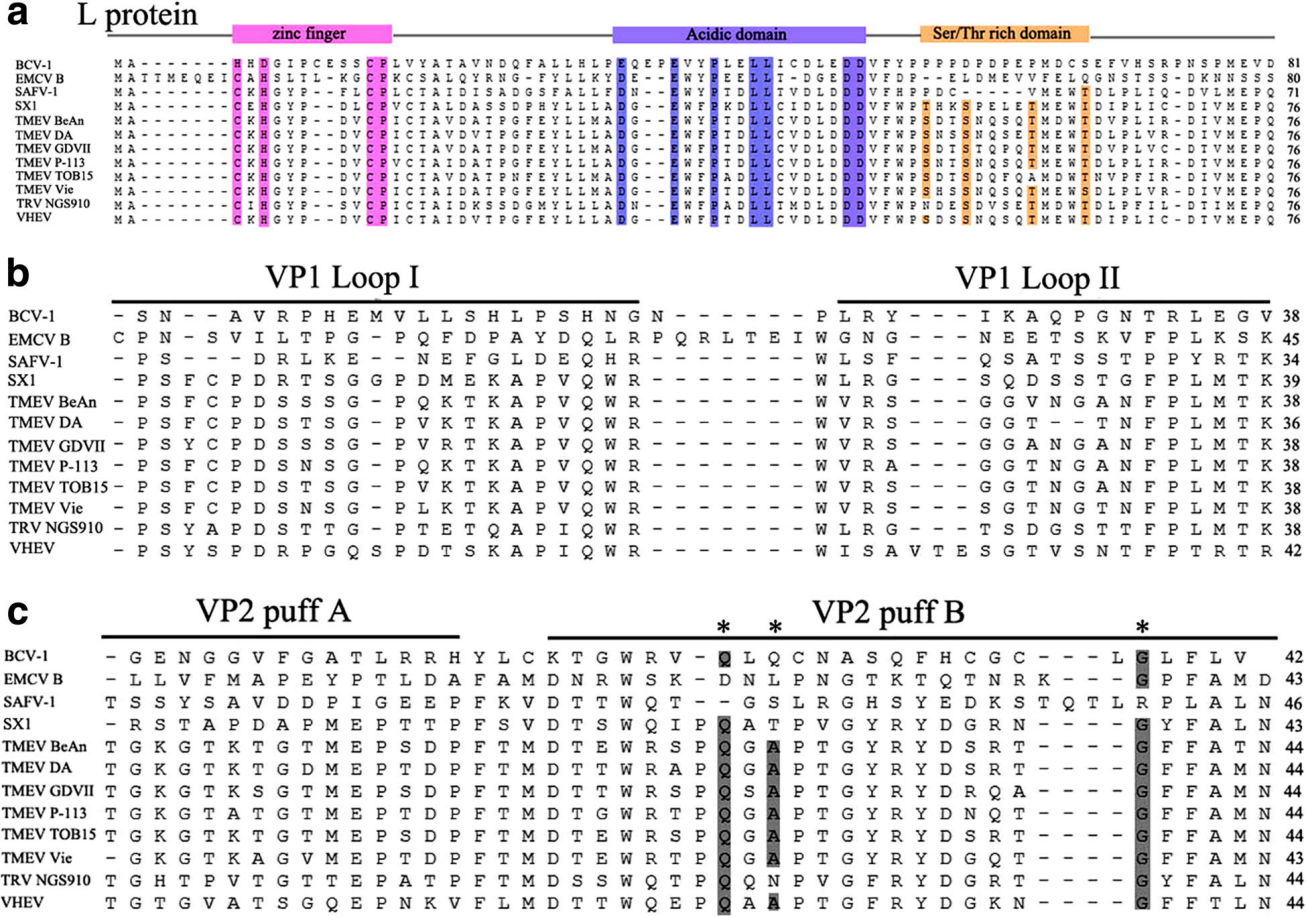
Alignment of amino acids of L protein, VP1 loop I and II, VP2 puff A and B among different cardioviruses. **a** Alignment of amino acids of L protein. The zinc finger (pink), acidic domain (blue), and Ser/Thr rich domain (brown) was shown. Two mutations in the Ser/Thr rich domain was found in SX1. **b, c** Alignment of amino acids of VP1 loops and VP2 puffs. Three key resides of VP2 puff B on the viral surface binding to host cells were marked gray and as asterisk. Low homology in VP1 loop II and an alternative residue “T” in VP2 puff B that is vital for sialic acid binding was found

We further analyzed the key proteins of SX1 which mainly related to antagonizing host immunity or being involved in host cell tropism and viral pathogenesis, including L protein [17], VP2 puffs A and B, and VP1 loops I and II [18]. The L protein is thought to have multiple functions including the regulation of the trafficking of interferon-regulatory proteins to the nucleus, binding to Ran GTPase and blocking the nuclear export of new mRNAs [17, 19]. Being similar to TMEV, SX1 encoded an L protein including three domains: an N-terminal atypical (CHCC) zinc finger, an acidic domain, and a C-terminal Ser/Thr-rich domain (Fig. 3a). Compared with other TMEVs, there were two mutations of Ser and Thr residues in the Ser/Thr-rich domain of SX1 (Fig. 3a), which is associated with the viral pathogenicity. Whether the mutations can affect viral pathogenicity remains to be addressed. In addition, a 156-aa L* protein was also encoded 13 nucleotides downstream of the start site of polyprotein (data not shown), which was thought to have a function in viral persistence because the mutant leading to early termination of L* protein inhibited TMEV persistence [20]. Comparing with other TMEVs and VHEVs, L* protein of SX1 shared the highest amino acid sequence identity (79%) with one VHEV strain which caused degenerative neurological disease of inhabitants of Siberia [21]. In cardioviruses, the surface of VP1 loops I and II, VP2 puffs A and B are exposed on the capsid surface and are thought to be involved in host cell tropism and viral pathogenesis [18]. Comparing with TMEVs and TRV NGS910 strain, although VP1 loop I of SX1 was more similar to TMEVs, VP1 loop II, VP2 puffs A and B of SX1 had higher amino acid sequence identities with TRV NGS910 strain (Fig. 3b and c). TMEVs VP2 puff B is believed to be responsible for sialic acid binding for viral entry, where three VP2 amino acids of puff B (Q^2161^, A^2163^, and G^2174^) within a positively charged area on the viral surface are vital for this binding [22]. Comparing with TMEVs, only two of the three residues in SX1 were the same to TMEVs (Fig. 3c). These data support our preceding conclusion in the phylogenetic analysis that SX1 is a novel strain with different epitopes and may have different pathogenicity.

To investigate the prevalence of SX1 in rats, we designed nested PCR primers targeting VP2 gene of *cardiovirus* by RT-PCR. Primer SX1 F1 (5’-GCCCATCGCGGAGAACACCC-3′) and SX1 R1 (5’-TGTCCAGGAGCTGGTCGGGG-3′) were used for the first round of PCR, and SX1 F2 (5’-CGGGGCTTTCTCCCACGTTCG-3′) and SX2 R2 (5′- CGTTTCGGCCGTCATAGCGGT-3′) for the second round, the expected length of amplicons was 308 bp. PCR screening results showed that 3 of the 40 fecal samples were positive with positive rate of 7.5% (3/40). The three positive samples were all obtained from rats collected in Gaogang. The specific PCR products were sequenced by Sanger method. Sequence alignment showed that the three 308 bp sequences shared > 98% nucleotide identities with each other, while shared < 79% nucleotide identities with other members of the genus, indicating the prevalence of a single strain in the wild rats in this area.

## Conclusions

In summary, we identified a novel cardiovirus in wild rats and characterized its complete genome. SX1 encodes a polyprotein including an L protein, four structural proteins and seven non-structural proteins, in addition, an L* protein is encoded 13 nucleotides downstream of the start site. Comparing with TMEVs and TRVs, many amino acids are different among them in loops of VP1 and puffs of VP2, both of those domains are vital for viral binding with host cells. The amino acid differences in those domains indicate that SX1 may have different pathogenic and antigenic properties. Phylogenetic analysis showed that the SX1 formed a separate branch that was distant from TMEVs and TRVs. The epidemiologic study suggested a single strain was prevalent in the wild rats in this area.

## Abbreviations

EMCV: Encephalomycarditis virus
SAFV: Saffold virus
TMEV: Theiler’s murine encephalomyelitis virus
TRV: Thera virus
VHEV: Vilyuisk human encephalomyelitis virus
